# “Crawl, walk, run”: A graded approach to integrating mental skills for psychological resilience in training for large-scale combat operations

**DOI:** 10.1080/08995605.2026.2612680

**Published:** 2026-03-02

**Authors:** Andreas Espetvedt Nordstrand, Haakon Gabrielsen Engen, Amy B. Adler

**Affiliations:** a Center for Military Mental Health, Joint Medical Forces, Norwegian Armed Forces, Oslo, Norway; b Department of Psychology, Norwegian University of Science and Technology (NTNU), Trondheim, Norway; c Department of Psychological Science, Northern Arizona University (NAU), Flagstaff, Arizona, USA; d Center for Military Psychiatry and Neuroscience, Walter Reed Army Institute of Research (WRAIR), Silver Spring, Maryland, USA

**Keywords:** Resilience, stress inoculation, combat, mental skills, zone of optimal arousal

## Abstract

Modern large-scale combat operations (LSCO) combine high-tech weapons such as drones and advanced sensors, with low-tech tactics like trench warfare and massed infantry assaults. The Russian invasion of Ukraine illustrates key LSCO features: limited cover and concealment, high casualty rates, and little opportunities for respite or recovery. Such conditions place extraordinary psychological demands on service members. Beyond preparing troops with tactical training, this environment requires explicit practice of mental skills under operational stress. This paper presents findings from the Operational Resilience Training (ORT) program, a U.S.-Norwegian collaboration designed to build mental readiness for LSCO. ORT has been delivered to Ukrainian service members rotating in and out of frontline duty. The course follows a “crawl, walk, run” progression: instruction (“crawl”), structured field practice (“walk”), and high-intensity simulations that test skills under duress (“run”). This approach allows trainees to internalize and apply mental skills such as controlled breathing, attention control, and peer support during the preparation, performance and recovery phases of a mission. Evaluation data indicate that ORT is well-received and leads to significant increases in confidence of abilities to help oneself and others manage combat stress. Anecdotal reports suggest that ORT techniques are being successfully applied in real combat settings by both novice and experienced personnel. Thus, the current paper describes ORT skills and implementation practices aimed at maintaining performance and well-being among troops in current and future LSCO environments.

**What is the public significance of this article?—** Modern large-scale combat operations combine advanced technologies with low-tech, high-exposure infantry tactics, creating an extraordinary psychological stress burden on service members, comparable to earlier inflection points such as the introduction of machine guns and tanks in World War I. This study shows how a structured resilience program, Operational Resilience Training (ORT), uses a graded “crawl, walk, run” approach to strengthen service members’ confidence in managing their own combat stress and that of others. The findings support integrating systematic psychological skills training into military doctrine, force preparation, and training policy as a core component of operational readiness.

Large-scale combat operations (LSCO) involve high-intensity, sustained warfare that challenges the physical, emotional, and cognitive limits of service members (Hoyt & Hein, 2021). Modern LSCO are increasingly defined by the asymmetric convergence of high-tech capabilities, such as drones, precision-guided munitions, and electronic warfare, with low-tech tactics like trench warfare and massed infantry assaults (Department of the Army, Training and Doctrine Command, G, 2024). In such environments, service members face continuous threats of lethal harm, the loss of teammates, witnessing the death and suffering of others, and exposure to prolonged operational stress without respite (e.g., Anyan et al., 2024). Resources like air support and rapid casualty evacuation, accessible in the previous global war on terror, are less likely to be available. These challenges can degrade performance and increase the risk of acute stress reactions (ASR) or longer-term psychological sequalae (e.g., Adler & Gutierrez, 2022; Britt et al., 2021; Hoge et al., 2014; Nordstrand et al., 2019; Taverniers et al., 2010a).

Preparing troops for LSCO will, therefore, not only require tactical and physical training but also training that increases psychological readiness (e.g., Huffman et al., 2025). Indeed, future conflicts will likely demand exceptional psychological resilience from service members at all levels. Even those in support or “non-combat” roles may find themselves under fire or handling combat-related stressors such as mass casualties or moral dilemmas in multi-domain battlefields. Given these parameters, it is important that militaries include effective, easily employable training in psychological resilience when preparing their service members for combat.

Psychological resilience in the military context refers to the capacity to withstand and bounce back from stress and adversity in war (e.g., Bonanno et al., 2011; Nindl et al., 2018; Nordstrand, Bøe, et al., 2024). Over the past two decades, militaries have invested in resilience-building initiatives amid evidence that formal training in mental skills can improve service members’ performance under stress (e.g., Bowles & Bates, 2010; Crouch et al., 2024; Foran et al., 2012; Nordstrand, Barger, et al., 2024; Tornero-Aguilera et al., 2024). Recent research in military populations supports the use of targeted training to enhance skills such as attentional control, stress regulation, and adaptive coping in order to improve service members’ performance and health in high-stress situations (Adler et al., 2015; Cacioppo et al., 2015; Doody et al., 2022).

Traditional training paradigms for combat stress adaptation typically involve exposing service members to realistic battlefield stressors during training exercises (Hourani et al., 2011; Taylor et al., 2011). The assumption is that experiencing stress in training will inoculate service members against the stress associated with real combat (e.g., Meichenbaum & Deffenbacher, 1988; Saunders et al., 1996). While developing “train as you fight” tactical training scenarios is necessary, service members may benefit from first learning practical skills in managing stress, before being exposed to highly demanding training.

In addition to building mental skills for psychological resilience, it is also important to consider how stressors are introduced into training scenarios aimed at practicing such skills. For example, it has long been suggested that the best structure for resilience training is through introducing stress in a graded format (e.g., Meichenbaum, 1993; Morgan et al., 2019). Despite these longstanding recommendations, militaries may find themselves skipping the skill-building component and, moreover, may fail to set up increasingly demanding scenarios that offer service members a chance to master these skills. This deliberate framework of skill building before exposure, as well as a graded format, is key, because if stress exposure in training is too intense or unstructured, the stress exposure may overwhelm trainees and prove counterproductive (e.g., Morgan et al., 2006). Conversely, if stress exposure is too controlled or artificial, it can fail to prepare service members adequately for the unpredictability of war (Morgan et al., 2019). Achieving the right balance is both a pedagogical and a scientific challenge. In this paper, we highlight an example of how one approach to resilience training, called Operational Resilience Training ([ORT] Iversen & Adler, 2023; Nordstrand, Engen, et al., 2024) can directly achieve this balance by integrating the practice of mental skills into increasingly stressful LSCO relevant scenarios in order to promote mastery and confidence.

## A resilience training program for LSCO: Operational resilience training (ORT)

ORT is a structured mental skills training program developed to enhance service members’ psychological resilience and stress management abilities in combat settings. Previous resilience training programs have been designed to train service members in mental skills using a variety of approaches, including training mental skills during basic combat training (Adler et al., 2015; Park et al., 2022), as part of a full-scale master resilience training program (Reivich et al., 2011), and following combat deployment (e.g., Battlemind; Adler et al., 2009; Castro et al., 2012). ORT builds on these foundational efforts and expands their impact through a focus on combat-related mental skills and integration into military scenario training for LSCO. The ORT program was co-developed by the Joint Medical Service of the Norwegian Armed Forces (JMSNAF) and the U.S. Army’s Walter Reed Army Institute of Research (WRAIR), leveraging WRAIR’s expertise in the field of resilience and military mental health (Adler & Gutierrez, 2022; Adler et al., 2015, 2021; Crouch et al., 2024; Mesias et al., 2024).

The war in Ukraine has provided a real-world crucible to test the effectiveness of ORT under LSCO conditions. The training was originally conducted in Norway as part of Operation Gungne, a Norwegian effort to train Ukrainian service members outside Ukraine. Later, it was expanded to other countries working with Ukraine as part of the multinational operation EU Military Assistance Mission Ukraine (EUMAM UA) (Nordstrand, Engen, et al., 2024). Throughout the program’s development, teams of Norwegian military mental health instructors (in partnership with U.S. subject-matter experts) have delivered ORT courses to Ukrainian combat medics and other frontline troops.

Launched in 2023, the program’s current iteration consists of 20 hours of training, conducted first in the classroom and then in practical field exercises, typically implemented over a three-day period. The skills taught during these three days of ORT training are subsequently integrated into a five-week combat medic course in order to reinforce learning and coping skills during demanding situations (e.g., battle drills, aid station evacuation, casualty field care).

The content of ORT is comprehensive, covering key domains of combat-related stress and coping (Iversen & Adler, 2023), and is continuously in development based on feedback from trainees, reports from the field, and program evaluation (see Table 1 for a description of the modules and an overview of mental skills). Early modules introduce the spectrum of combat stressors, from minor daily hassles to acute traumatic events (Anyan et al., 2024) and emphasize that stress reactions exist on a continuum of normal to severe (Ram et al., 2023). Trainees learn to recognize where a service member falls on this stress reaction continuum, from the “blue zone” of optimal performance to the “red zone” of combat ineffectiveness, and to tailor interventions accordingly. Moreover, the trainees are taught foundational skills for frontline mental health providers such as active listening and communication techniques like closed-loop communication and rules for interpersonal conflict (Nordstrand, Engen, et al., 2024).

**Table 1.** Operational resilience training (ORT) modules and integration in stress lane training.

Module	Sample topics	Stress lane scenario
Fundamentals of Stress and Resilience	**Recognizing Combat stressors**; The Stress Reaction Continuum; **Stress Bucket**	Yes
Forward Military Mental Health	Assessment; Key Mental Health Symptoms and Field-Appropriate Interventions, including Suicide Risk; Active Listening, Cognitive Behavioral Techniques; Steps for Rest and Recovery	No
Operational Stress Control	**Deliberate Breathing**; **Self-Talk/Buddy Talk**; **Attention Control Techniques**; Cue Words; **Grounding**; **Types of After Action Reviews**; Active Appreciation and Gratitude	Yes
iCOVER	**Peer-to-Peer Procedure for Managing Acute Stress Reactions**	Yes
Cohesion	**Rules of Engagement for Interpersonal Conflict**; **Closed Loop Communication**; Addressing Social Withdrawal; Encouraging Social Inclusion of Newcomers; Building Trust; Creating Unit Culture	Yes
Frontline Grief	Self-care, Connection, Expectation Management, Acknowledge	No
Mental Skills for Managing Human Remains	**Exposure control**, **Attention Control, Arousal Control**, Sustainment	Yes

^**a**^A “yes” response indicates that some or all of the skills in a given ORT module was deliberately integrated into the stress lane scenario.

**Bolded** items are specifically prompted by training cadre during the scenarios.

Following these knowledge modules are instructions in combat stress control techniques. The ORT techniques cover a range of skills including the “What’s Important Now” (WIN) technique for attention control, effective strategies for self-talk and buddy-talk, and emotion regulation techniques like grounding. These techniques are divided into categories of phase-specific skills, with skills being labeled as particularly applicable for use in the preparation (e.g., visualization, worry postponement), performance (e.g., grounding, control the controllables), and recovery (e.g., progressive muscle relaxation [PMR], active appreciation) phases of a mission.

Another segment addresses the recognition and management of ASR in fellow service members, a relatively common occurrence on the battlefield (Adler & Gutierrez, 2022). ASRs may occur when an individual is so overwhelmed by stress that they stop functioning for a period of time (Adler & Gutierrez, 2022), displaying a behavioral pattern of freezing, dissociation or agitation (World Health Organization, 2018). Trainees practice identifying signs of ASRs and learn a specific protocol known as “iCOVER” for stabilizing someone experiencing the condition, much as they would apply medical first aid to a physical injury (e.g., Adler et al., 2020).

The name iCOVER is an acronym for a series of steps: **I**dentify individuals having an ASR; **C**onnect to the individual using voice, visual cues and touch; **O**ffer commitment by letting the individual know they are not alone; **V**erify facts by asking simple questions that prompts the individual to cognitively engage; **E**stablish order of events to ground the individual in time by explaining what has happened, what is happening and what will happen; and **R**equest action by directing the individual to take part in a purposeful activity like loading ammunition, carrying the wounded, or scanning for threats to counter a sense of helplessness.

Additional ORT modules emphasize other relevant resilience factors such as the importance of sleep and restoration, methods for managing cumulative stress and fatigue, and strategies for fostering unit cohesion and buddy support as a buffer against combat stress. By addressing such topics, ORT prepares frontline caregivers not only to stabilize others’ acute reactions but also to cope with their own emotional responses to battlefield stressors. Notably, ORT also includes content on handling stressors particular to high-intensity combat, with modules covering frontline grief management and mental skills for handling human remains.

## A rationale for skills-based resilience training

Traditionally, militaries have recognized the need to expose troops to stressful situations in training to prepare them for the shock of combat (Taylor et al., 2011). The underlying assumption is that the more realistic and high-pressure the training is, the tougher and more resilient service members will become (Hourani et al., 2011; Sudom et al., 2014). However, emerging evidence suggests that this traditional paradigm has important limitations (e.g., Fikretoglu et al., 2019; Skeffington et al., 2013; Sun et al., 2024; Teigen, 1994).

In particular, exposure to high stress in training without the necessary skills can risk overloading the trainee’s capacity to cope, leading to outcomes opposite to those intended (Bulmer et al., 2022; Feigel et al., 2025; Forse et al., 2025; Nindl et al., 2018; Tait et al., 2024). For example, research has evidenced that this level of intense stress during military training can acutely impair tactically important capabilities such as memory, judgment, and fine motor skills (Morgan et al., 2006; Passi et al., 2022). Such poor calibration of intensity can also lead to excessive arousal or confusion that degrades skill development, undermining service member confidence (Taverniers et al., 2010a, 2010b, 2013).

These findings align well with cognitive overload theory (e.g., Sweller, 2011), in which the introduction of too many novel stressors at once can overwhelm working memory and learning. In such cases, the service member is not so much “inoculated” against stress as overwhelmed, which can impair learning and even create negative associations with the task (Lieberman et al., 2005; Taverniers et al., 2011). In line with learned helplessness theory (Seligman, 1972), a service member subjected to non-deliberate stress inoculation efforts might simply learn that certain situations feel unmanageable, rather than learn how to effectively cope in those circumstances. This learned helplessness would likely foster passivity and avoidance rather than adaptive coping, precisely the opposite of what resilience training aims to achieve. Accordingly, rather than asking, “have we stressed the service members enough to harden them?” a more useful question may be “have we taught the service members how to handle increasing stress effectively?” Taking this perspective, stress exposure is a tool for reinforcing skills and confidence, not an end in itself.

## Calibrating stress exposure: The optimal arousal for learning and stress mastery

In the context of training service members for LSCO, calibrated stress exposure means dialing the difficulty and stress intensity in training scenarios to stay near an optimal zone of arousal for learning, given the trainees’ current skill level. This “zone of optimal arousal” refers to a level of physiological and psychological arousal that maximizes performance, based on the inverted-U relationship between arousal and task efficiency first described by Yerkes and Dodson (1908). The concept is corroborated by studies that indicate ways in which training can be scaffolded to improve the service member’s capacity to respond to stress. For example, high elevations in heart rate (e.g., above ~170 bpm) correlate with worse shooting performance (Hoffman & Street, 1992). Training that teaches service members to modulate their physiological arousal (e.g., through breathing, focus, and confidence built by practice) can directly translate to better battlefield performance (e.g., Adler et al., 2015; Sun et al., 2024; Tornero-Aguilera et al., 2024).

This calibrated stress exposure is an underlying paradigm for ORT scenario training. Trainees are first taught skills to control their arousal level, so they have a toolkit for dealing with the various stressors in demanding tactical environments (Table 1). Then, trainees are exposed to incrementally increasing challenges that provide them with opportunities to train and master these skills under simulated combat conditions.

The approach can be illustrated by a scenario used in ORT, where instructors use a decibel meter on their smartphone to gauge the noise level of teams training to evacuate a field hospital under artillery simulation. The rational applied is that teams under higher stress tend to shout more, indicating rising arousal that can impair performance (Langer et al., 2022; Lee et al., 2023; Sörensen et al., 2022; Wang et al., 2025). If the decibel reading crosses a certain threshold (e.g., 75 decibel, indicating the team is getting too loud and frantic), instructors can pause the action briefly to remind trainees to employ their mental skills (e.g., “check in with yourself, and assess your arousal levels,” “breathe, communicate, and think: What’s Important Now?”), prompting participants to reduce their arousal back into the optimal range. This kind of real-time feedback loop in training is an experiential way to demonstrate to trainees that there is a link between stress and performance. The activity also reinforces the point that the trainees have the mental tools to control their stress reactions, thus increasing their confidence in their ability to cope. Over time, trainees may internalize and later aim for the feeling of being at optimal arousal: an engaged, focused state that is neither too lax nor too anxious. The goal is to enable service members to self-correct under fire (e.g., recognize when panic is creeping in and deliberately calm themselves to maintain effectiveness). Importantly, the decibel gauge was only used when training medical teams in field hospital evacuation, since keeping sound to a minimum may not be applicable in all combat scenarios. However, this use of a decibel gauge is an example of how easily fielded indicators of physiological arousal can be implemented in LSCO training to calibrate stress intensity.

Accordingly, instructors designing such LSCO training scenarios should consider: what is the optimal arousal level for their training audience? If a scenario is yielding only confusion and gross errors, it might be a sign that the stress level overshot the intended arousal; the instructor may need to dial stress levels back or better prepare the trainees with skills. Conversely, if a scenario is too easy and everyone breezes through without elevated arousal or any mistakes, the scenario likely wasn’t stressful enough to induce adaptation, and the next iteration should be designed to be more challenging. Calibrating stress is an iterative practice that often requires instructors to read trainees’ behavior, check in with trainees’ regularly, and adjust in stride. Objective measures of physiological stress (e.g., decibel gauge; heart rate) or simple observations of body language, communication and cohesion can guide these adjustments. Moreover, this approach enables trainees to learn that stress is not a binary state (calm vs. panic) but a continuum they can navigate and influence. This insight will often replace the fear of stress with a sense of familiarity and control (Jensen et al., 2020; Sörensen et al., 2022). Thus, when trainees step into the unpredictable stress of real combat, they can be better able to stay at arousal levels where they can think clearly, act decisively, and fulfill their mission.

## Gradual immersion: The “crawl – walk – run” framework

A way to operationalize the graded format of stress exposure in resilience training is to use the “crawl – walk – run” framework (Table 2). This method entails setting up the training in a way the enables service members to become confident in the use of mental skills that promote resilience. In military training doctrine, “crawl – walk – run” describes a phased approach where skills are introduced at a basic level (“crawl”), then practiced with more complexity (“walk”), and finally applied under realistic, demanding conditions (“run”). Applying this structure for mental skills training, a novice trainee would not be thrown straight into the most demanding scenario to teach them skills; instead, beginners would start practicing skills in a low-risk, low-demand environment. As their skills and confidence grow, trainees are challenged to tackle more difficult scenarios (e.g., Liu, 2022). The aim is not to coddle trainees or avoid stress, but to ensure they internalize proper techniques (e.g., controlled breathing, cognitive reframing, team communication protocols) when the stakes and psychological demands are low, so that these techniques become second nature, and are then more readily available when the stakes are high (Haji et al., 2016; Shuggi et al., 2017). This framework is effective in everything from marksmanship to battle drills (Guadagnoli & Lee, 2004; Marraffino et al., 2021), and it is equally applicable to training mental skills for psychological resilience (Jensen et al., 2020).

**Table 2.** Examples from the operational resilience training (ORT) “stress lane” case library.

Scenario number	Training phase	Name	Scenario	Stressors	Learning objectives
1	“Crawl”	iCOVER Basics.	Small unit, one service member in acute stress. Instructor guided.	Calm environment. Actor with simulated ASR (unresponsive, disoriented)	Practice iCOVER protocol steps.
2	“Walk”	iCOVER Basics with Buddy Oversight.	Repeat of S-1 with peer observer instead of instructor.	Field environment. Casualty in ASR. Actor with simulated ASR (agitated). Trainee now more autonomous (peer guides if needed)	Reinforce iCOVER skills with less direct coaching. Build confidence in executing steps in order from memory Introduce peer After Action Review (AAR).
3	“Run” (easy)	Contact Drill with iCOVER.	Firefight with one team member becoming ASR casualty (frozen)	Field environment. Simulated gunfire. Physical exertion. Actor with ASR.	Apply Care Under Fire. Perform emergency medical skills under stress. Execute iCOVER during combat. Practice communication.
4	“Run” (moderate)	Squad Ambush with Multiple Casualties.	Patrol ambushed, one team-member wounded from shrapnel and suffering from ASR.	Intense simulated gunfire. Simulated grenades. Team coordination. Single ASR casualty with physical wounds.	Maintain communication. Tactical withdrawal while treating casualty. Apply iCOVER with minimal supervision under duress. Decision-making under stress.
5	“Run” (advanced)	Complex Attack with Mass Casualty.	Multiple simultaneous casualties due to gunfire and shrapnel.	Multi-directional threat. Intense simulated gunfire. Simulated artillery shelling (C4). Drones harassing team.	Triage under pressure. Arousal control. Attention Control. Communication. Adaptive teamwork.
6	“Run” (advanced) Moral	Moral Dilemma under Time Pressure.	Having to abort first aid on pregnant civilian casualty.	Moral and emotional stressors. Time and social pressures.	Apply “What’s Important Now” (WIN). Apply” Control the Controllables.” Emotional distancing.
7	Following “Run” (Advanced) Scenarios	Post-Battle Relaxation Coaching.	Guided recovery after action.	No external stressors.	Self-guided progressive muscle relaxation. AAR. Transition from high to low arousal.

In the context of ORT training, “crawl” might involve simple classroom exercises. Using iCOVER training as an example: “crawl” involves classroom instruction on the steps of iCOVER with a slow-paced practical exercise, while “walk” is a moderate simulation using the technique in a basic tactical scenario with liberal time limits and a moderately agitated casualty. Finally, “run” would be a full-blown field exercise; however, such “run” scenarios will further vary in intensity. In ORT these gradations of “run” field exercises are formally split in to a range from easy and moderate to advanced, in order to ensure optimal calibration of stress intensity throughout the training cycle.

As an illustration of how the varying stressors culminate together in a “run” advanced stress lane, trainees in such scenarios may be asked to perform iCOVER on an ASR casualty in a smoke-filled area with people screaming, while an “enemy” role-player attacks them with simulated munitions (e.g., training grenades). By this “run” stage, the trainees will have already internalized the basic skills during the “crawl” and “walk” phase. The chaotic scenario now tests their ability to execute under pressure, reinforcing that they can trust the techniques when it counts.

In summary, the “crawl – walk – run” framework encapsulates a pedagogy of gradual, guided immersion. It encourages instructors to carefully calibrate the introduction of stressors, ensuring that trainees are technically and mentally ready to handle the next level of difficulty.

## Designing realistic stress scenarios with targeted outcomes

Building on the principles of gradual exposure and optimal arousal, the next task in designing resilience training is to operationalize these principles into realistic scenarios. Military training has long used “lanes” (scripted scenario exercises) for various skills, with different cases consisting of scenarios designed to test a particular skill. In ORT training the same framework is applied: a “stress lane” is a scenario lane with embedded psychological challenges (Figure 1) set up as series of scenarios or cases that aim to provide the trainees with opportunities to practice different ORT skills. Instructors, acting as “scenario designers,” should consider several dimensions while respecting safety protocols:

**Figure 1.**
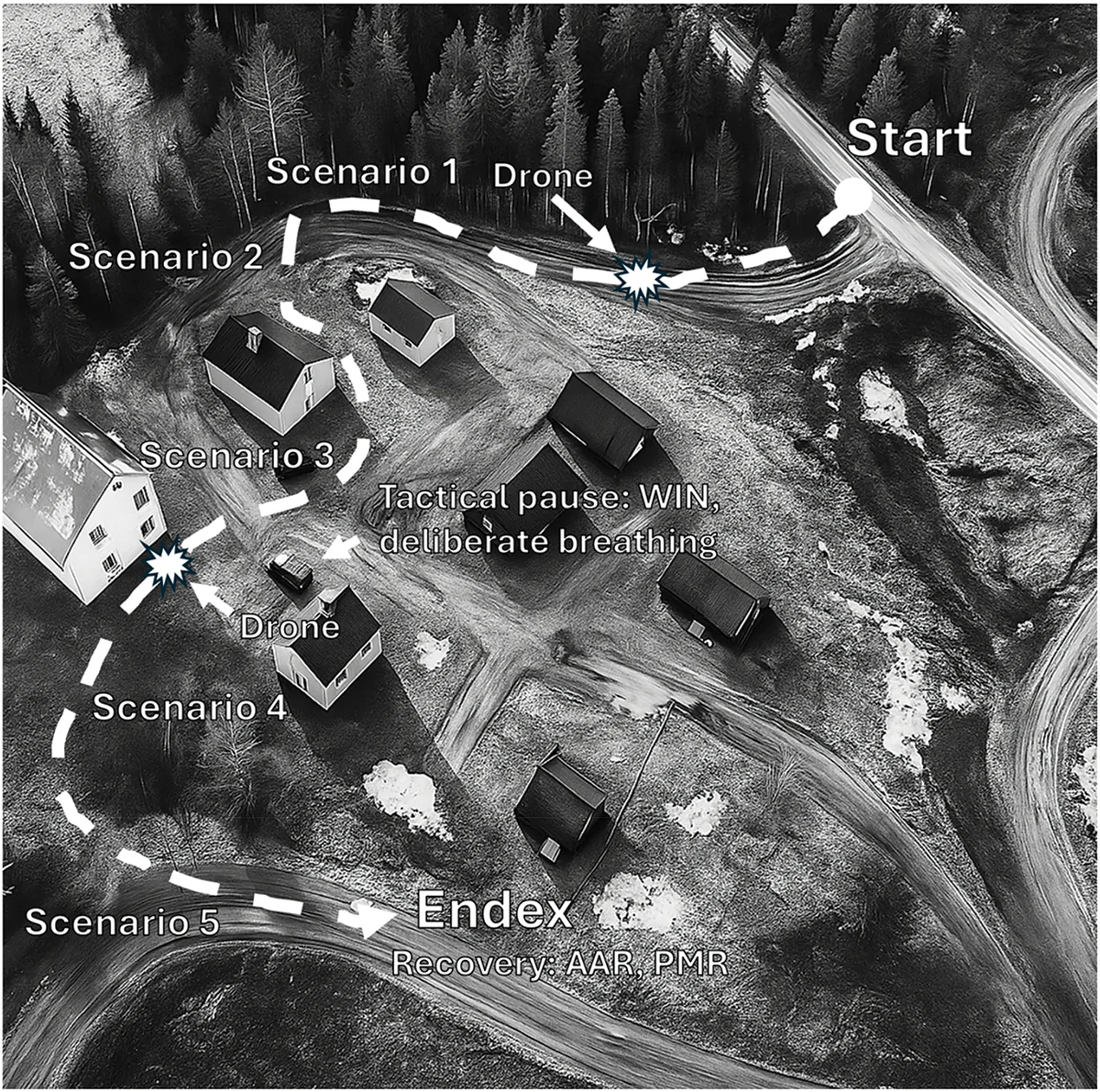
Operational resilience training (ORT) Stress lane example.

At scenario 1, the team encounters an unconscious torture victim in the woods. They hear the Russian national anthem playing and encounter the smell of rotten meat and blood. They see torture instruments on the ground. They are also threatened by “enemy” drones that are flying overhead and dropping grenades; the team must leave the torture victim. At scenario 2, the team sees an Improvised Explosive Device (IED) that is clearly visible and must reroute. At scenario 3, the team encounters a pregnant civilian who is scared, in pain and unable to walk. The cadre instructor (“squad leader”) tells the team they need to shift focus, keep going, and complete their assigned mission. After departing that scene, the team is instructed to take a tactical pause. The cadre instructor (“squad leader”) reminds the team to apply the What’s Important Now (WIN) technique and use deliberate breathing. Leading into scenario 4, the team is attacked by another drone before encountering a soldier who is hanging from a tree. The soldier is unconscious but alive and needs to be rescued. At scenario 5, the team encounters the gruesome remains of a child in the woods. One of the team members (the translator) enacts an acute stress reaction (ASR) in order to prompt appropriate intervention by the trainees. When the team completes the stress lane (marked “Endex” in the map), the cadre instructor (“squad leader”) guides the team through an after-action review (AAR) and a progressive muscle relaxation (PMR) exercise.

- **Training Objective**: What mental skill or resilient behavior is this scenario meant to target? For example, consider targeting effective communication under duress, maintaining focus amid distractions, ethical decision-making under pressure, recovering from startle/fear response, or other behavior.
- **Scenario Context**: What is the narrative or tactical setting? For example, consider creating a patrol ambush, casualty evacuation, urban operation with civilians, chemical weapons attack or other potential LSCO scenarios.
- **Training Phase**: Is this scenario meant to be a “crawl,” “walk,” or “run” opportunity? Taking into account trainee proficiency levels in using the mental skills, align the scenario to create the intended difficulty in terms of complexity and stress intensity.
- **Stressors Employed**: Which stress elements will be present? Consider elements across a variety of dimensions, including sensory overload, time pressure, complex multi-tasking, physical load, social/evaluative stress, moral dilemmas, personal threat/fear and uncertainty (see Table 3 for a description of stressors and examples).

**Table 3.** *S*tressors for developing resilience training scenarios to promote mastery.

Stressor	Example
Sensory overload	Loud noises (e.g., simulated artillery, gunfire, explosions, screaming), chaotic visuals (e.g., flashing lights, smoke, darkness), smells (e.g., blood, diesel).
Time pressure	Time constraints on tasks to induce a sense of urgency (e.g., multiple casualties bleeding out).
Complex multitasking	Requiring attention to many things at once (e.g., radio messages coming in during a firefight, handling triage decisions).
Physical load	Fatigue from prior exercises, heavy lifting (e.g., carrying a casualty), environmental challenges (e.g., heat, cold), hunger or sleep deprivation.
Social/evaluative stress	Presence of observers or VIPs, performance being evaluated or ranked, peer pressure.
Moral dilemmas	Scenarios forcing a hard choice, such as prioritizing whom to save, encountering civilian casualties, or rules of engagement (ROE) ambiguities that create ethical stress.
Personal threat/fear	Simulated life-threatening situations (e.g., direct fire, near-misses), or role-players acting as aggressive enemy combatants confronting the trainee.
Uncertainty	Limiting information about events in the scenario or deliberately changing events in a way that conflicts with previously given information (e.g., having critical equipment like a radio malfunction unexpectedly).
Intensity	Having role-players reflect a heightened emotional state (e.g., acute grief reactions to simulated death or injury in friendly forces).

Each of these stressors target a different psychological and physiological effect. For example, loud noise and chaos are designed to target fear and confusion. Moral dilemmas are designed to induce a sense of guilt or anxiety; physical exhaustion is designed to induce irritability or cognitive slowing; and social evaluation is designed to trigger performance anxiety. By anticipating these reactions, instructors can prompt participants to use specific coping responses (like using an attention control model like “What’s important now (WIN)” to handle moral stress, or self-talk to handle performance anxiety). Levels of arousal can be monitored via observation of task performance, behavior, speech volume, a decibel gauge or other easily scalable indicator.

When creating a training scenario, instructors should have preplanned moments to inject or remove stressors and should continually assess the trainees’ use of appropriate mental skills. For instance, an instructor might deliberately introduce a moral stress-element (e.g., by having a civilian cry for help when giving aid would run counter to mission objectives and constraints) and then assess if the service member applies prior training (e.g., by recalling ORT skills to not become paralyzed by guilt and indecision). Alternatively, an instructor might simulate equipment failure at a critical juncture to induce frustration and then coach the trainee to self-regulate and problem-solve by means of the right techniques.

Finally, at the end of a stress lane, there needs to be an after-action review (AAR). This AAR should incorporate a debrief that focuses on the skills targeted in the different scenarios: What did the trainee feel during their peak stress level? Was the arousal too high or too low? Did they recognize the stress levels of their teammates? How did they manage this stress? What techniques were employed? What worked or failed? By utilizing AAR in this manner after completing a stress lane, instructors can facilitate reflective learning and mastery of the trained skills, as well as enhancing their own awareness of the trainees’ training progression (e.g., Moldjord & Hybertsen, 2015). In addition, this way of evaluating a training exercise using AAR frames psychological resilience as set of trainable mental skills, not a reflection of a trainee’s character.

The ORT “stress lane” case library used in Operation Gungne exemplifies many of these design considerations. Table 2 provides a synthesized excerpt of this library, illustrating several scenario types across the “crawl – walk – run” spectrum, the stressors they involve, and their key learning objectives in terms of practicing particular mental skills. The table demonstrates how scenarios grow in complexity and stress, from basic skills training to full-scale trench warfare simulations with drone threats, consistent with the LSCO environment.

Initially ORT-library scenarios have a low intensity and are intended to reinforce basic skills. By scenarios 4 and 5, trainees are exposed to squad-level engagements with multiple simultaneous demands, mirroring the fog of war (see Table 3 for examples). Instructors controlling these exercises must ensure safety and that training goals are met. For example, scenario 5 recommends having multiple instructors and role-players to create the effect of incoming indirect fire, drone threats, etc., to really push the unit’s limits. In these advanced “run” scenarios, maintaining optimal arousal will be challenging as the scenarios are intended to allow trainees to test the limits of their abilities. Accordingly, some trainees will start to become overloaded, perhaps team communication is disrupted, or someone is temporarily stuck in indecision. Thus, the instructor-led AAR will be crucial to maximize learning after the stress lane. Useful questions in the subsequent AAR can therefore be: “What techniques could you have used when you felt overwhelmed?” and “What would you do next time to stay in the game?” These scenarios serve as a capstone for ORT training, revealing how far the trainees have progressed in terms of managing combat and operational stress, and where more work must be done.

## Broadening resilience training to include moral and recovery domains

Unfortunately, a total-war battlespace will inevitably contain many moral challenges (e.g., Nordstrand et al., 2025), and the tension between humanitarian impulses and operational necessity mirrors real-world ethical dilemmas that service members frequently face in such wars. These harsh realities underscore the importance of also training mental skills applicable for morally challenging scenarios when preparing service members for LSCO (Thompson & Jetly, 2014). The inclusion of scenario 6 (Moral Dilemma under Time Pressure) is therefore particularly noteworthy as it introduces an explicit moral stressor. In this scenario, the service member encounters a wounded pregnant civilian who cries out and begs not to be left behind. This situation places trainees in a psychologically challenging situation that readily evokes stress and negative feelings. Training such moral scenarios can be easy to overlook in military training as they do not directly relate to tactical skills. However, anger, shame, guilt and inner conflict, feelings commonly associated with moral challenges in war (Nordstrand, Anyan et al., 2024), can drain the mental resources of services members and thus impede performance. When integrating such scenarios, trainees are prompted to respond to the moral distress using the attention control techniques like WIN and “Control the Controllables,” as well as supportive self-talk and buddy-talk. Furthermore, morally challenging scenarios give trainees an opportunity to see the utility of ORT skills also in situations that do not necessarily relate directly to combat actions. Ultimately, integrating moral stressors into resilience training reinforces the broader utility of ORT: trainees see that attention control and self-regulation techniques are just as essential when confronting moral tension as when managing physical threats. This experience can thus expand their understanding of resilience as a capability that spans both tactical and ethical complexities.

Finally, scenario 7 (Post-Battle Relaxation Coaching) in the table represents a different, but crucial aspect of resilience: recovery and reset. After putting trainees through high-stress scenarios, it is important to train the “cool-down” as well. Such scenarios can involve trainees practicing leading each other in relaxation techniques, such as PMR, since learning how to deliberately relax and lower one’s physiological arousal after a battle is a skill that can reduce cumulative stress. The “cool-down” phase will be particularly relevant in LSCO scenarios where cumulative stressors are likely to take a toll on troops (e.g., Britt et al., 2021; Nordstrand et al., 2019). During ORT scenario 7, instructors observed that trainees who were initially apprehensive (e.g., expressing that PMR would be too “touchy-feely”) came to appreciate how the technique has an immediate stress regulatory effect. Accordingly, including PMR in training sends a message: resilience is not only about charging into stress but also about recovering afterward. Accordingly, designing a resilience training pipeline should span the full cycle of encounters with high-stress situations: preparation, performance under stress, and post-stress recovery.

## Trainee feedback and evaluation of ORT

As the ORT program matured, pre- and post-surveys were developed to assess training acceptability and changes in participant confidence in using ORT skills (Wilhelmsen et al., 2025). Each item was rated on a 5-point scale from strongly disagree to strongly agree. These brief surveys were designed to be linked over time using a personalized code in order to keep responses anonymous. In the second year of the ORT program, all participants in the ORT course were invited to complete two surveys that could be linked over time. Of the 278 trainees available, 273 completed the pre-training survey. While 255 completed the post-training survey, difficulties with missing and incomplete linking codes resulted in only 145 (52.2%) responses that could be linked over time.

Immediately after the training, over 90% of participants reported that ORT was useful and relevant to their role, and that they intended to use the resilience skills they learned (Wilhelmsen et al., 2025). Moreover, almost all (99%) participants rated the training as important for them and their unit, and 95% rated the overall course satisfaction as “good” or “very good.” Such approval ratings underscore the degree to which experienced troops find value in the ORT skills.

Interestingly, one might assume that veteran service members (70% of the sample) would be jaded, or that “toughness” learned in battle would diminish the perceived value of a resilience training course. However, the opposite was observed, with the most experienced service members enthusiastically embracing ORT, and many reporting that the material filled critical gaps in their skill set that had not been covered in their prior training.

When survey responses were compared from pre- to post-training, service members showed improved confidence regardless of their combat experience. On average, there was a 41% increase in service members’ confidence in their ability to help manage a unit member’s stress reactions in combat, and a 26% increase in confidence in managing their own stress (Wilhelmsen et al., 2025). These improvements indicate that ORT not only imparts knowledge but also builds self-efficacy, a factor linked to better performance under stress (Delahaij & Van Dam, 2017). In a 4-week follow-up survey, these new confidence levels were sustained (Wilhelmsen et al., 2025). Although performance outcomes in combat could not be tracked due to operational realities, this sustained boost in confidence suggests ORT instilled lasting improvements in skills and mind-set.

Beyond the survey data, we also had informal feedback from Ukrainian trainees. This feedback came from routine classroom conversations with participants during the ORT courses and also from follow-up contact after the courses. During the course, participants frequently commented that the ORT skills were needed and immediately applicable for addressing the stress of being deployed to the Ukrainian frontlines.

After the course, some trainees also reached back to the ORT instructors with anecdotes they wanted to share about their experiences in the field. For example, they described using active listening and breathing techniques to help calm fellow service members while hunkering down during frequent shelling in the trenches (A. E., Nordstrand & C.L., Gjerstad., personal communication, September 28, 2024). One combat medic highlighted that he introduced the ORT materials to his fellow medics, with the result that the medics became more proactive in checking on their unit members’ state of mind and intervening early when they saw signs of overarousal or exhaustion (A. E., Nordstrand & C.L., Gjerstad., personal communication, September 28, 2024). Finally, anecdotal reports from trainees also indicate that the ORT training reduced the intensity of difficult emotions in morally complex situations. Specifically, trainees have reached out to report that the ORT training enabled them to maintain attentional control in morally challenging frontline triage situations, by focusing on what they could influence rather than what would be possible in a hospital setting (A. E., Nordstrand & T. Irgens., personal communication, April 20, 2025). While we recognize that this kind of informal feedback from in-class discussions and post-course communication was not systematically obtained or analyzed, such anecdotes add an important dimension to understanding how ORT can be useful for troops at the frontlines.

Consistent with the positive feedback from the survey and other sources, it is also interesting to note that several Ukrainian combat medics instructors trained in ORTs at sites in Europe have begun integrating ORT into combat medic courses they now conduct close to the front lines. Moreover, the National Guard of Ukraine (NGU) has recently mandated 9 hours of ORT training for all their approximately 100,000 service members (Holovina, 2025; Osypenko, 2025). Overall, such implementation efforts, by a military organization that is hard-pressed in their training time priorities, further suggest that ORT provides substantive value to the war fighting capabilities of service members engaged in LSCO.

## Limitations to implementation

Although this paper offers relatively universal principles for constructing scenarios that train mental skills for psychological resilience, several limitations merit consideration. First, the implementation of resilience training integrated into tactical scenarios necessitates specific personnel competencies and material resources. Designing detailed, immersive scenarios requires time, appropriate expertise (i.e., tactical and psychological competencies), materials (e.g., simulated casualties, pyrotechnics, space, and equipment) and coordination. The complex and realistic ORT scenarios described in the current paper were developed and executed by mental health and resilience experts working in collaboration with personnel specializing in military tactical training. This example highlights the need for cross-disciplinary input; mental health professionals aiming to develop similar scenarios may need to partner with military subject matter experts.

Second, scalability remains a challenge because military systems typically operate with high training throughput (Nindl et al., 2018). The challenge is in part that it is difficult to calibrate individualized stress exposure for large military formations (e.g., entire brigades). One way to address scalability is through integrating ORT-type stress scenario training into parts of existing military activities, such as field maneuvers. This integration could be done by embedding uniformed or civilian mental health providers (e.g., psychologists, psychiatric nurses, social workers) in the military training cadre that is planning and executing the field maneuvers in order to incorporate a deliberate resilience training dimension. An added benefit of integrating mental health providers as resilience trainers in field maneuvers could be to reinforce their role as a prevention workforce in the military. Furthermore, assembling a collection of “crawl – walk – run” scenarios, including training videos that illustrate the key principles, can also help streamline uptake and innovation. Initial “crawl” exercises could be provided via a hybrid format (both online and live).

Finally, while ORT has shown promising results, its empirical foundation is still limited. There is currently no systematic evidence on long-term behavioral or performance outcomes, nor on generalizability across different military units or national contexts.

## Future directions

Future research should continue to refine resilience training methodologies and evaluate their long-term impact on operational performance and mental health. Expanding these programs to include diverse military populations, including those in support roles exposed to high cognitive and emotional demands, will improve the ecological validity of findings.

There is also a pressing need to develop reliable field metrics to assess psychological resilience in real-time. Devices that indicate physiological arousal may be useful in providing immediate feedback to trainees acquiring resilience skills and their training cadre. These kinds of devices should be simple, easily interpreted, and scalable. In addition, alternative metrics may need to be considered such as stress-performance metrics (e.g., communication efficacy under duress) and artificial intelligence-based tools for real-time adjustment of scenario difficulty, which may also enhance training precision. Another fruitful area for investigation is the interplay between resilience skills, unit cohesion, and leadership style. These factors may distinctly interact with one another under different levels of operational stress.

Ultimately, while anecdotal evidence, confidence gains, and user feedback from Ukrainian trainees, are highly encouraging, more rigorous and comparative evaluations will be required to build a robust evidence base. Future research should test ORT within a longitudinal framework and, if feasible, assess ORT using naturalistic experiments that allow for comparison to units that do not get ORT. This kind of evaluation would build the empirical evidence necessary for determining whether ORT resilience skills are used during deployment and whether they promote better performance, sustained functioning and post-deployment mental health, all essential components of determining efficacy.

## Conclusion

As militaries worldwide prepare for the possibility of large-scale, long-term, and high-intensity warfare, the psychological dimension of warfighting must be prioritized alongside traditional competencies such as marksmanship, physical fitness, and tactical skill. The findings summarized in this paper support the inclusion of structured graduated resilience training as a core component of military readiness for LSCO. Approaches that emphasize maximum realism without first ensuring skill mastery risk overwhelming trainees, and may be counterproductive. By contrast, training that employs a “crawl – walk – run” pedagogy and calibrates stress exposure to optimize learning and performance fosters confidence and adaptability under pressure. Reports from Ukrainian combat medics trained in ORT suggest that this approach has immediate real-world relevance. Moreover, psychological resilience training can be embedded in military training culture through small, regular practice. For example, mental skills tips can be added to routine briefings, arousal checks can be incorporated into range exercises, and AARs can be conducted that address recovery strategies alongside tactical outcomes. The ORT program illustrates that such efforts are both feasible and likely impactful. In the end, perhaps the most compelling evidence lies in the human stories of Ukrainian medics who use ORT skills to great effect in LSCO. These accounts offer a vision of resilience training not just as nice to have, but as an operational requirement in the modern LSCO battlespace.

## Data Availability

Data and materials related to this article can be accessed by contacting the authors directly.
